# Cancer incidence and mortality after a first-ever venous thrombosis: a cohort study in northern Sweden

**DOI:** 10.1186/s12959-024-00646-z

**Published:** 2024-08-21

**Authors:** Lovisa Hägg, Felicia Ehrs, Marcus Lind, Magdalena Johansson

**Affiliations:** Department of Public Health and Clinical Medicine, Skellefteå Research Unit, Umeå University, Umeå, Sweden

**Keywords:** Venous thromboembolism, Pulmonary embolism, Deep vein thrombosis, Cancer, Mortality, Cohort study

## Abstract

**Background:**

Venous thromboembolism (VTE) has a high mortality rate and can be the first manifestation of cancer. We investigated the incidence of cancer after first-ever VTE and the association between VTE and all-cause mortality.

**Methods:**

A Swedish cohort study that included 105,997 participants without previous cancer who underwent a health examination from 1985–2014 was conducted. Manually validated first-ever VTE events, incident cancer according to the Swedish cancer registry, and mortality were registered. Participants were followed until September 5, 2014.

**Results:**

The mean age at inclusion was 46.2 years, and 50.3% of participants were female. We identified 1303 persons in the cohort with a VTE and no previous cancer. Among these, 179 (13.7%) were diagnosed with cancer after the VTE event, resulting in a cancer incidence of 26.4 (95% CI 22.8–30.6) cases per 1000 person-years. The incidence was highest during the first 6 months after the VTE. In the study population, VTE was associated with an increased risk of cancer (HR 1.95 [95% CI 1.67–2.29] in a multivariable model). VTE was also associated with an increased risk of death (HR 6.30 [95% CI 5.82–6.81]) in a multivariable model). There was an interaction between sex and VTE in relation to both risk of cancer and mortality, with a stronger association in women.

**Conclusions:**

The incidence of cancer is high after first-ever VTE, especially close to the VTE event. VTE seems to be a stronger risk marker in women than in men for both cancer and death.

**Supplementary Information:**

The online version contains supplementary material available at 10.1186/s12959-024-00646-z.

## Background

The incidence of venous thromboembolism (VTE) is approximately 167/100,000 person-years, and as many as 20–30% of VTE cases can be associated with cancer at the time of diagnosis [1, 2]. The mechanisms linking cancer to venous thrombosis are suggested to include cancer cell activation of the coagulation system, vessel wall injury, and hemodynamic alterations to blood flow due to tumor growth [3, 4]. The correlation between VTE and cancer was described as early as in the 1860s [5]. Since then, many studies have confirmed that cancer substantially increases the risk of VTE [6–9]. In addition, a diagnosis of VTE can precede a diagnosis of cancer [10, 11].

Previous observational studies have shown that the risk of finding cancer is elevated after a VTE diagnosis and, according to some studies, the risk is highest during the first 6–12 months after the VTE event [12, 13]. However, due to inherent misclassification of VTE diagnosis in registers, it is difficult to accurately estimate the risk of cancer after VTE without validation of the VTE diagnosis [14]. Despite the strong correlation between cancer and VTE, extensive screening for cancer at the time of VTE diagnosis has not been proven to be beneficial [15, 16]. With time, the incidence of cancer after VTE may change, and knowledge of the accurate cancer incidence at different time intervals from the VTE event could be clinically useful for estimating the risk of cancer after VTE in individual patients.

In addition to the association with morbidity, VTE is also associated with increased mortality [17, 18]. A decreasing frequency of autopsies and the possibility of VTE either being the primary cause of death or contributing to mortality in patients with other comorbidities, such as cancer, makes it difficult to determine the number of VTE-related deaths using only death certificates or hospital discharge diagnostic codes [19]. Reliable data on the current mortality in patients with VTE could be important in the assessment of the effects of any new health care measures aiming to prevent VTE-related deaths. This means that it is useful to explore VTE-associated mortality in a population-based setting.

We performed a longitudinal observational study in northern Sweden to investigate the prognosis for persons with a validated first-ever VTE diagnosis. The aim of our study was to determine the incidence of cancer at different intervals after a first-ever VTE. We also aimed to investigate the association between VTE and all-cause mortality.

## Methods

### Study population

**Fig. 1:**
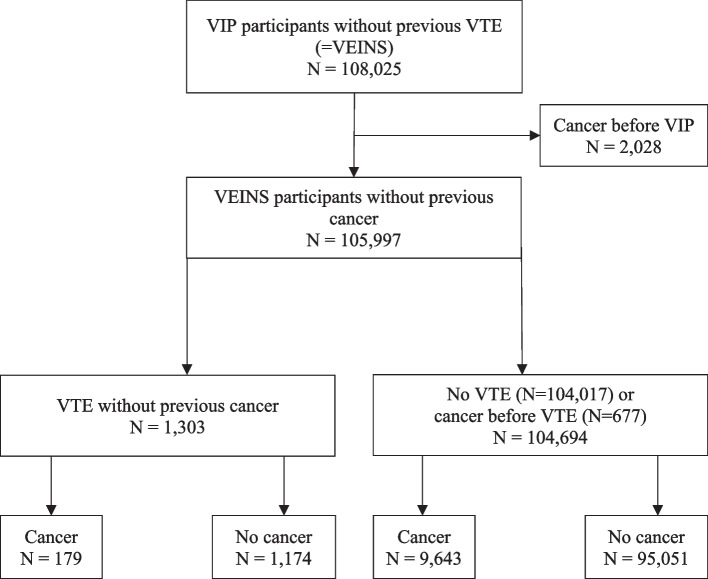
Flowchart of the study population. VIP, Västerbotten Intervention Programme; VTE, venous thromboembolism; VEINS, Venous thromboembolism in northern Sweden

Identification of the study population is illustrated in Fig. 1. Our study participants were derived from the Västerbotten Intervention Programme (VIP), an ongoing population-based health examination program in northern Sweden that started in 1985. This survey has been described in detail previously and included persons aged 30–60 years [20]. VIP participants without VTE before the health examination who also gave consent for research were included in a previously formed cohort called “Venous thromboembolism in northern Sweden” (VEINS). These participants were followed until a first-ever VTE, emigration, death, or end of study on September 5, 2014. The VTE events in the VEINS cohort were found through a diagnosis registry search for specific International Classification of Diseases (ICD) diagnostic codes indicating a potential VTE event. The diagnosis search was performed in registers for inpatient care years 1985–2014 and in the register for specialized outpatient care from its founding in 2001 onwards. In addition, we searched the local registry of primary health care diagnoses. All potential VTE events were manually validated by scrutinizing medical records, as well as autopsy and radiology reports [21]. In the present study, we included VEINS participants without cancer before the health examination. These participants were followed until a diagnosis of cancer, emigration, death, or end of study (i.e., September 5, 2014). Cancer incidence was calculated for participants with a first-ever VTE during the study period but without cancer before the VTE event. The ICD 8, ICD 9 and ICD 10 codes used to identify potential VTE events and to exclude the presence of VTE events that had occurred before study inclusion are listed in Appendix Table 1.

### Variable detection and classification

We classified the VTE events according to location as pulmonary embolism, lower extremity deep vein thrombosis (DVT), or other VTE (i.e., upper extremity DVT, abdominal VTE, or sinus thrombosis). Individuals presenting with thrombosis at multiple locations were classified hierarchically as pulmonary embolism followed by lower extremity DVT and last other location. Information was collected on provoking factors other than cancer at the time of VTE diagnosis for participants with a VTE event in 2006 and later by scrutinizing electronic medical records. For patients with earlier VTE events, this data was not available. We defined the VTE event as provoked if any of the following risk factors were present within 60 days prior to the VTE: hospitalization, surgical intervention, immobilization > 48 h, recent trauma, cast therapy, travel > 8 h, central venous line, hormone therapy, pregnancy, or postpartum (within 60 days of delivery).

Cancer diagnoses were obtained from the nationwide Swedish Cancer Register. The register started in 1958 and, because it is mandatory for health care providers to report new cancer diagnoses to the register, the coverage rate is very high, up to 96% [22]. From the register, we collected the date of cancer diagnosis and cancer type. Cancer types were categorized into 19 different groups (see Appendix, Table 2). Data about smoking, body mass index (BMI), and education level were collected from questionnaires administered at the baseline health examination. Smokers were categorized as “ever smokers” or “never smokers”, and education level was categorized as “secondary school or below”, in contrast to “above secondary school”. Weight and height were measured without shoes and in light clothing and BMI calculated. Mortality data were collected from the Swedish population register using the unique personal identity number assigned to each Swedish resident.

### Statistical analysis and ethics

The baseline characteristics were calculated using descriptive statistics (numbers, percentages, means, and standard deviations). For participants with a first-ever VTE and without cancer before the VTE event, incidence of cancer per 1000 person-years was calculated at different times from the VTE event and stratified by sex, VTE location, and presence of provoking factors. To compare baseline characteristics between participants who did and did not develop VTE during follow-up, the chi-squared test and the t-test were used.

The association between first-ever VTE and cancer was analyzed using multivariable Cox proportional hazards regression. VTE was entered as a time-dependent covariate. Follow-up started at the VIP health examination. Persons with VTE during follow-up contributed VTE-free follow-up time until the VTE event, and then VTE-exposed follow-up time after the VTE event. A univariable model was used, as well as a model adjusted for age and sex and a model adjusted for age, sex, BMI, smoking, and education level. Analyses were carried out for the whole population and stratified by sex.

We also calculated the association between VTE and all-cause mortality using Cox proportional hazards regression. Analyses were carried out using a univariable model, a model adjusted for age and sex, and a model adjusted for age, sex, BMI, smoking, education level, and cancer. Cancer and VTE were entered as time-dependent covariates. Analyses were carried out for the whole population and stratified by sex. We also performed multiplicative interaction analyses for the sex-specific associations between VTE and cancer and for the sex-specific association between VTE and mortality.

The proportional hazards assumption was tested graphically by examining plots of the log-minus-log of the survival function versus the survival time, with separate curves for different levels of categorical covariates. For continuous variables, plots of smoothed, scaled Schoenfeld residuals versus survival time were examined. Proportional hazards were assumed for all covariates. Calculations were performed using STATA version 14 (Stata Corporation, College Station, TX, USA).

## Results

A total of 105,977 VEINS participants without a cancer diagnosis before the health examination were included in the current study and followed for a total of 1,427,349 person-years. Baseline data for the study population are provided in Table 1. The mean age was 46.2 (Standard deviation 9.2) years and 50.3% of the population was female. Baseline characteristics for participants that did and did not develop VTE can be found in the Appendix, Table 3. During follow-up, 9822 study participants were diagnosed with cancer (5092 males, 4730 females). The incidence of cancer in the whole study population was 6.88 (95% confidence interval [CI] 6.75–7.02) cases per 1000 person-years of follow-up. In men, the incidence of cancer was 7.33 (95% CI 7.14–7.54); in women, the incidence was 6.45 (95% CI 6.27–6.64) cases per 1000 person-years.

**Table 1:** Baseline characteristics of the study population at first health examination (*n* = 105,997)

Age, years	46.2 (9.2)
Female sex	53,302 (50.3%)
Smoking	
Never smoker	51,176 (49.2%)
Ever smoker	52,828 (50.8%)
Education level	
Secondary school or below	75,141 (72.2%)
Above secondary school	28,990 (27.8%)
BMI, kg/m^2^	25.8 (4.2)

Data are reported as n (%) or mean (standard deviation)

*BMI* Body mass index

In our total study population of 105,997 individuals, we identified 1980 participants with a verified first-ever VTE event during follow-up. These participants were followed for a total of 1,471,713.5 person-years resulting in an incidence of VTE of 1.3 cases per 1000 person-years (95% CI 1.3–1.4). Among the participants with a first-ever VTE, 1303 participants did not have cancer before the VTE. The mean age among these 1303 participants was 63 years, and 57% were men. The mean duration of follow-up from the VTE event to end of study was 5.2 years (median 3.9 years), and the mean time from the VTE event to cancer diagnosis was 3.3 years (median 1.1 years). Among the participants with a first VTE, but no previous cancer, 179 (13.4%) were diagnosed with cancer after the VTE, resulting in an incidence of 26.4 (95% CI 22.8–30.6) cancer cases per 1000 person-years (Table 2). During VTE-free follow-up time, 9643 cases of cancer occurred during a total follow-up time of 1,420,569 person-years, resulting in an incidence of 6.79 (95% CI 6.65–6.92) cases per 1000 person-years. The incidence of cancer after VTE was 28.8 (95% CI 23.1–35.8) cases per 1000 person-years in women and 24.7 (95% CI 20.3–30.1) cases per 1000 person-years in men. In participants with lower extremity DVT, the incidence of cancer was 24.3 (95% CI 19.8 – 29.9) cases per 1000 person-years, and in participants with pulmonary embolism it was 26.3 (20.8 – 33.3) cases per 1000 person-years.

**Table 2:** Incidence of cancer in persons with venous thromboembolism without previous cancer

		Study participants (n)	Cancer during follow-up (n)	Follow-up time, PY	Incidence per 1000 PY (95% CI)
All		1303	179	6780	26.4 (22.8 – 30.6)
Sex	Women	556	81	2817	28.8 (23.1– 35.8)
	Men	747	98	3963	24.7 (20.3 – 30.1)
VTE location	Pulmonary embolism	615	70	2660	26.3 (20.8 – 33.3)
	Lower extremity DVT	611	91	3742	24.3 (19.8 – 29.9)
	Other VTE^a^	77	18	379	47.5 (29.9–75.4)
Presence of provoking factors^b^	Unprovoked VTE	470	48	1505	31.9 (24.0 – 42.3)
	Provoked VTE	396	28	1187	23.6 (16.3 – 34.2)

*CI* Confidence interval, *PY* Person-years, *DVT* Deep venous thrombosis, *VTE* Venous thromboembolism

^a^Upper extremity DVT, abdominal VTE, and sinus thrombosis

^b^Persons diagnosed with VTE in the years 2006–2014 with any of the following risk factors present within 60 days before the VTE event: hospitalization, surgical intervention, immobilization > 48 h, trauma, cast therapy, travel > 8 h, central venous line, hormone therapy, pregnancy or postpartum (within 60 days of delivery)

Among the 1303 participants with VTE but without previous cancer, 866 were diagnosed with VTE between 2006 and 2014, allowing us to extract data on risk factors for VTE at the time of the VTE event. Among these participants, 396 (45.7%) had a provoked VTE event (provoking factor other than cancer). The most common risk factor for VTE was hospitalization (*n* = 289, 33.4%). The incidence of cancer was 23.6 (95% CI 16.3–34.2) cases per 1000 person-years in participants with provoked VTE and 31.9 (95% CI 24.0–42.3) cases per 1000 person-years in participants with unprovoked VTE.

**Fig. 2:**
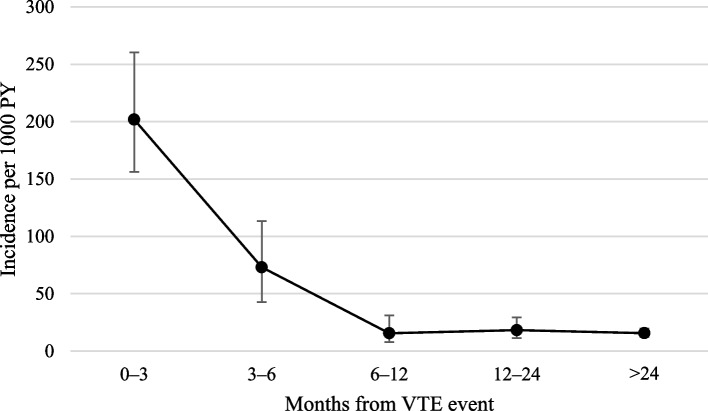
Incidence of cancer at different time intervals from first-ever VTE. Incidence shown with 95% confidence intervals. VTE, venous thromboembolism; PY, person-years

Table 3 shows the incidence of cancer stratified by follow-up time. The cancer incidence was 201.8 (95% CI 156.3–260.4) cases per 1000 person-years during the first 3 months of follow-up; 3–6 months after the VTE event, the incidence of cancer was 73.1 (95% CI 47.2–113.3), and it rapidly declined after 6 months from the VTE event and stabilized at around 15 cases per 1000 person-years (Fig. 2).

**Table 3:** Incidence of cancer per 1000 PY at different time intervals after first-ever VTE

	Participants/cases (n)	Follow-up, years	Incidence (95% CI)
0–3 months	1303/59	292	201.8 (156.3 – 260.4)
3–6 months	1127/20	274	73.1 (47.2 – 113.3)
6–12 months	1065/8	513	15.6 (7.8 – 31.2)
12–24 months	991/17	928	18.3 (11.4 – 29.5)
> 24 months	860/75	4773	15.7 (12.5 – 19.7)

*VTE* Venous thromboembolism, *CI* Confidence interval, *PY* Person-years

The associations between VTE and risk of a subsequent cancer diagnosis are presented in Table 4. Participants with VTE had an approximately twofold higher hazard of cancer compared to participants without VTE (hazard ratio [HR] 1.95 [95% CI 1.67–2.29] in a multivariable model). The HR for cancer was higher in women than in men, and a multiplicative interaction analysis showed a significant interaction (*p* = 0.02) between VTE and sex in relation to risk of cancer in a multivariable model.

**Table 4:** Associations between a first venous thromboembolism (VTE) event and risk of cancer (*n* = 105,997)

	All (*n* = 105,997)		Men (*n* = 52,695)		Women (*n* = 53,302)	
	Crude HR	Adjusted HR^a^	Crude HR	Adjusted HR^b^	Crude HR	Adjusted HR^b^
First VTE	2.94 (2.54–3.41)	1.95 (1.67–2.29)	2.41 (1.97–2.95)	1.43 (1.16–1.77)	3.68 (2.95–4.59)	2.81 (2.23–3.54)
Male sex	1.15 (1.11–1.20)	1.17 (1.12–1.22)	-	-	-	-
Age	1.08 (1.08–1.08)	1.08 (1.08–1.08)	1.10 (1.10–1.10)	1.10 (1.10–1.10)	1.06 (1.06–1.06)	1.06 (1.05–1.06)
Daily smoking	1.25 (1.20–1.30)	1.22 (1.17–1.27)	1.38 (1.31–1.47)	1.19 (1.12–1.26)	1.11 (1.04–1.17)	1.19 (1.12–1.27)
Education up to secondary school	1.17 (1.12–1.23)	0.94 (0.89–0.99)	1.14 (1.06–1.22)	0.96 (0.89–1.03)	1.18 (1.11–1.27)	0.95 (0.88–1.02)
BMI, per standard deviation	1.16 (1.14–1.18)	1.03 (1.01–1.06)	1.12 (1.08–1.15)	0.99 (0.95–1.02)	1.18 (1.15–1.21)	1.08 (1.05–1.11)

All patients were free of cancer and VTE at inclusion. VTE was treated as a time-dependent covariate in the Cox proportional hazards regression analysis

Associations are shown as hazard ratios with 95% confidence intervals in parentheses

*HR* Hazard ratio, *BMI* Body mass index

^a^Adjusted for age, sex, venous thromboembolism, BMI, smoking, education level

^b^Adjusted for age, venous thromboembolism, BMI, smoking, education level

During the study period, 3112 (5.8%) women and 4514 (8.6%) men died. During VTE-free follow-up time of 1,471,714 person-years, 6814 deaths occurred, resulting in an incidence of 4.63 (95% CI 4.52–4.74) deaths per 1000 person-years. During follow-up time after a VTE event, 812 deaths occurred during a total follow-up time of 8539 person-years, resulting in an incidence of 95.09 (95% CI 88.77–101.86) deaths per 1000 person-years. In Table 5, the associations between a VTE event and risk of death are presented, stratified by sex. According to the fully adjusted model, persons with a first-ever VTE had an approximately sixfold higher hazard of mortality compared to patients without VTE (HR 6.30 [95% CI 5.82–6.81]). The HR was higher in women than in men (HR 8.06 [95% CI 7.19–9.04] and HR 5.13 [95% CI 4.60–5.73], respectively). A multiplicative interaction analysis showed a significant interaction (*p* < 0.001) between VTE and sex in relation to risk of death.

**Table 5:** Associations between a first venous thromboembolism (VTE) event and risk of death (*n* = 105,997)

	All (*n* = 105,997)		Men (*n* = 52,695)		Women (*n* = 53,302)	
	Crude HR	Adjusted HR^a^	Crude HR	Adjusted HR^b^	Crude HR	Adjusted HR^b^
First VTE	13.33 (12.38–14.37)	6.30 (5.82–6.81)	9.72 (8.76–10.77)	5.13 (4.60–5.73)	19.06 (17.12–21.21)	8.06 (7.19–9.04)
Cancer	15.58 (14.85–16.35)	9.21 (8.74–9.71)	13.53 (12.69–14.43)	7.54 (7.03–8.09)	19.09 (17.73–20.55)	12.42 (11.47–13.45)
Male sex	1.56 (1.49–1.64)	1.38 (1.32–1.45)	-	-	-	-
Age	1.12 (1.12–1.12)	1.09 (1.09–1.09)	1.12 (1.12–1.12)	1.09 (1.08–1.09)	1.12 (1.11–1.12)	1.10 (1.09–1.11)
Daily smoking	1.57 (1.49–1.64)	1.44 (1.37–1.51)	1.76 (1.65–1.88)	1.43 (1.33–1.52)	1.30 (1.21–1.40)	1.51 (1.40–1.63)
Education up to secondary school	1.94 (1.81–2.08)	1.36 (1.27–1.47)	1.64 (1.50–1.80)	1.29 (1.18–1.42)	2.16 (1.94–2.41)	1.45 (1.29–1.61)
BMI, per standard deviation	1.29 (1.26–1.32)	1.08 (1.06–1.11)	1.29 (1.25–1.33)	1.15 (1.11–1.19)	1.27 (1.24–1.31)	1.00 (0.96–1.03)

All patients were free of cancer and VTE at inclusion. Cancer and VTE were treated as time-dependent covariates in the Cox proportional hazards regression analysis

Associations are shown as hazard ratios with 95% confidence intervals in parentheses

*HR* Hazard ratio, *BMI* Body mass index

^a^Adjusted for age, sex, venous thromboembolism, BMI, smoking, education level, and cancer

^b^Adjusted for age, venous thromboembolism, BMI, smoking, education level, and cancer

The most common cancer types to be diagnosed after a VTE event were prostate cancer (*n* = 33) and colorectal cancer (*n* = 19). The associations between VTE and different cancer types are found in the Appendix Table 2. For example, in a model adjusted for age, sex, BMI, smoking, and education level, the HRs for the association between VTE and cancer were 5.41 (95% CI 2.84–10.34) for pancreatic cancer, 7.64 (95% CI 3.34–17.47) for ovarian cancer, and 7.64 (95% CI 4.10–14.26) for cancer with unknown primary tumor.

## Discussion

We found that individuals with a first-ever VTE event have a high incidence of subsequent cancer compared to individuals without a VTE event. Our findings are in line with previous studies. Nordstrom et al. studied patients undergoing phlebography in Malmö, Sweden, in 1994 and found that a VTE diagnosis was associated with a significantly higher frequency of malignancy during the first 6 months of follow-up [23]. A meta-analysis in 2008 found a threefold increased risk of occult cancer in patients with VTE [24]. In a population-based cohort study, Sun et al. investigated the cancer risk in 27,751 patients with unprovoked VTE and 110,409 matched comparison cases without VTE in Taiwan [12]. They found an overall adjusted HR of 2.3 (95% CI 2.16–2.37) for cancer in persons with unprovoked VTE, which is comparable to our finding of an adjusted HR of 1.95 (95% CI 1.67–2.29) for cancer in participants with a first-ever VTE.

In our study, the cancer incidence was highest during the first 6 months after the VTE event and then declined markedly (Fig. 2). Previous studies of cancer risk after a VTE event have had diverging results when investigating the risk over a longer time period. For example, Douketis et al. studied the long-term risk of cancer in patients with a first episode of VTE and showed a yearly risk of new cancer of 1–2% [25]. In contrast to our study, the risk seemed to be uniform over time. Baron et al. also showed an elevated risk of cancer for more than 10 years after a VTE event in Swedish patients admitted to the hospital for VTE between 1965 and 1983 [26]. Other studies have demonstrated a slight but significantly elevated cancer risk after VTE for up to 2 years or longer [12, 27]. In a large Danish study, Sørensen et al. evaluated the association between VTE and subsequent cancer, showing an excess risk of cancer the first year after the thrombotic event; however, after the first year, the risk of cancer was not significantly elevated compared to what could be expected from national cancer incidence rates [28]. The diverging results regarding cancer risk after VTE over a longer time period is likely explained by differences in study design, control groups, and cohort sizes. We suggest that, if an increased cancer risk persists in individuals with VTE years after the VTE event, it is modest at most.

In our cohort, the incidence of cancer after a VTE event was similar for persons with lower extremity DVT and pulmonary embolism. This finding is supported by Sørensen et al. and Jara-Palomares et al. [28, 29]. With these findings as the basis, it is reasonable to consider pulmonary embolism and thrombosis of the lower extremity as different manifestations of the same disease since the risk of future cancer seems to be mainly independent of VTE location. This is in accordance with it being common for persons with lower extremity DVTs to have concurrent pulmonary embolisms and vice versa [30–32].

We also found that patients with provoked VTE had a numerically lower incidence of cancer than patients with an unprovoked VTE. Most previous studies of the risk of cancer after VTE were performed in patients with unprovoked (idiopathic) VTE. Thus, less is known about the risk of cancer after a provoked VTE event. A meta-analysis from 2001 reported that most studies on provoked VTE have demonstrated cancer incidences similar to that of the general population [33]. In a more recent study, the annual risk for new cancer was higher after unprovoked VTE than after provoked VTE, but the risk was significantly elevated in both groups compared to the general population [25]. As there are no established consensus definitions of primary/idiopathic and secondary/provoked VTE, the risk factors used to define provoked VTE in different studies vary. This may explain some of the differences in study results.

In our study, the effect of VTE on the risk of cancer was pronounced for ovarian cancer (HR 7.64 [95% CI 3.34–17.47]) and for cancer with unknown primary tumor/metastases (HR 7.64 [95% CI 4.10–14.26]). The latter is in line with the results of a previous study by Sorensen et al. in which 40% of the patients with a cancer diagnosis had metastatic disease within 1 year after hospitalization for thromboembolism [28].

Women had a higher risk of cancer after a VTE than men. This result was unexpected because previous observational studies showed no significant difference in cancer risk after an unprovoked first-ever VTE between men and women [12, 28, 34]. Sanden et al. even found that men with VTE had a higher risk of cancer than women [13]. In our population, the baseline incidence of cancer was lower in women than in men, which could contribute to the relatively larger effect of VTE incidence on the future risk of cancer in women.

In both sexes, VTE was associated with increased all-cause mortality. In our fully adjusted model, patients with VTE had a sixfold higher hazard of mortality than patients without VTE. This indicates that VTE is an important condition to study further and find possible preventive measures and better treatments to hopefully reduce the excess mortality after VTE. Unexpectedly, we found a stronger association between VTE and all-cause mortality in women compared to men. Most previous studies on sex differences regarding VTE-related mortality have investigated short-term mortality after VTE events, with diverging results [35–37]. In a study from 2014, Blanco-Molina et al. studied sex differences in 47,499 patients enrolled in the RIETE study. There was similar all-cause mortality in men and women (HR 1.03 [95% CI 0.97–1.09]), but women had a higher hazard of fatal pulmonary embolism (HR 1.24 [95% CI 1.04–1.47]) [37]. In an American study from 2022, sex differences in VTE-related long-term outcomes were studied among patients with thrombosis in a tertiary care center. They found no sex difference in overall mortality but an increased risk of VTE-related mortality in females compared to males [38]. The underlying cause of our finding of a stronger association between VTE and mortality in women is unknown, but it is possible that the VTE-related mortality in women was higher than in men in the population as a whole, whereas the mortality due to other causes was higher in men. Some residual confounding factors that we have been unable to adjust for are also likely; for example, women with VTE could have more chronic conditions (other than cancer) than men. We were also unable to adjust for cancer stage, a factor known to be associated with both VTE and mortality.

### Strengths and limitations

A major strength of our study is the population-based design in a well-defined area of northern Sweden and the inclusion of both inpatients and outpatients with VTE. This, together with manual validation of all VTE cases, gives us an unselected, but well-defined study population. Without the manual validation of VTE cases, there is a considerable risk of including other diagnoses due to wrong ICD code (e.g., thrombophlebitis or leg edema), which could dilute the study results. We included a wide range of ICD codes to identify as many potential VTE events as possible. However, we cannot exclude the possibility that some VTE events were missed due to incorrect coding in the medical records.

Our study participants were aged 30 to 60 years at inclusion. The mean follow-up time was 13.47 years (median 14.84 years). To some extent this limits our power regarding potential associations in the oldest age-group. Our study cohort was not designed to identify the youngest VTE-patients and the findings in the present study are not applicable to this group.

Our observational study design makes it impossible to conclude causality. As we were only able to adjust for cancer but no other comorbidities, portions of the association between VTE and death could be explained by an unknown confounding factor. Another limitation of our study is the lack of data about the stage of cancer at diagnosis. We also lack data on provoking factors for VTE in patients diagnosed before 2006. Due to this, analyses taking provoking factors into account are limited in size and to a specific time interval.

There is a possible delay in reporting cancer diagnoses to the cancer registry. Some of the cancer diagnoses that are registered early after a VTE event could have been identified before or at the same time as the thrombotic event, incorrectly inflating the incidence of cancer during the first months of follow-up. However, while being aware of this limitation, one can conclude that there is an association between the two diagnoses close to the VTE event, and that the incidence of cancer declines rapidly after 6 months of follow-up. As the correlation between VTE and cancer has been well known for a long time, it is also possible that surveillance bias is introduced because it is probable that physicians, to some extent, scan for symptoms indicative of cancer in VTE patients. Hemminki et al. described this bias in their 2017 paper and stated that it is difficult to avoid in observational studies in a clinical setting [39]. To the best of our knowledge, extensive cancer screening after VTE was neither recommended nor commonly performed in Västerbotten, Sweden, by the time of our study.

## Conclusion

For patients with a first-ever VTE, the incidence of subsequent cancer is high, especially during the first 6 months after the VTE event. VTE is also associated with increased all-cause mortality. The associations between VTE and subsequent cancer, and between VTE and death seem to be stronger for women than for men.

## Supplementary Information

Supplementary Material 1.

## Data Availability

Due to restrictions in the ethics approval, the datasets for the current study cannot be made publicly available.

## Abbreviations

VTE: Venous thromboembolism
VIP: Västerbotten intervention programme
VEINS: Venous thromboembolism in northern Sweden
ICD: International Classification of Diseases
DVT: deep vein thrombosis
BMI: Body mass index
CI: Confidence interval
HR: Hazard ratio
