# Supplementary material for: Cancer incidence and mortality after a first-ever venous thrombosis: a cohort study in northern Sweden
Source: Thromb J. 2024 Aug 21;22:77. doi: 10.1186/s12959-024-00646-z (PMC11337770; doi:10.1186/s12959-024-00646-z)
Supplement: Supplementary file 1 — Supplementary Material 1. [file 12959_2024_646_MOESM1_ESM.docx]

**Appendix table 1** The ICD 8, ICD 9 and ICD 10 codes used to identify VTE events

| ICD 8 | 321.00; 321.09; 426.02; 426.08; 426.09; 438.00; 438.99; 440.20; 450.01; 450.02; 450.03, 450.09; 451.00; 451.98; 451.99; 452.99; 453.00; 453.99; 631.00; 631.10; 631.11; 631.20; 631.30; 634.50; 634.99; 642.00; 642.20; 643.00; 643.20; 644.00; 644.20; 671.00; 671.01; 671.02; 671.08; 671.09; 673.00; 673.10; 673.98; 673.99; 674.99; 677.98; 677.99. |
| --- | --- |
| ICD 9 | 325X; 415A; 415B; 416A; 416B; 416W; 416X; 437G; 444C; 451A; 451B; 451C; 451W; 451X; 452X; 453A; 453B; 453C; 453D; 453W; 453X; 557A; 634G; 634H; 634W; 635G; 635H; 635W; 636G; 636H; 636W; 637G; 637H; 637W; 638G; 638H; 638W; 639G; 639W; 639X; 671C; 671D; 671E; 671F; 671W; 671X; 673A; 673B; 673C; 673D; 673W; 674A |
| ICD 10 | I26.0; I26.9; I27.8; I27.9; I67.6; I74.3; I80.0; I80.1; I80.2; I80.3; I80.8; I80.9; I81.9; I82.0; I82.1; I82.2; I82.3; I82.8; I82.9; K55.0; O08.2; O08.7; O22.2; O22.3; O22.5; O22.8; O22.9; O87.0; O87.1; O87.2; O87.3; O87.8; O87.9; O88.0; O88.1; O88.2; O88.3; O88.8 |

VTE, venous thromboembolism

ICD, International Classification of Diseases

**Appendix table 2** Incidence of different cancer types in persons with a first-ever VTE-event and associations between a first-ever venous thromboembolic event and the risks of different cancer types.

|  |  | Cancer during follow-up (n) | Follow-up time (PY) | Incidence per 1,000 PY (95% CI) | Adjusted HR* |
| --- | --- | --- | --- | --- | --- |
| Head and neck | VTE | 3 | 6780 | 0.44 (0.14–1.37) | 2.01 (0.64–6.33) |
|  | No VTE | 197 | 1,420,569 | 0.14 (0.12–0.16) |  |
| Esophagus and stomach | VTE | 10 | 6780 | 1.47 (0.79–2.74) | 3.93 (2.00–7.74) |
|  | No VTE | 267 | 1,420,569 | 0.19 (0.17–0.21) |  |
| Colorectum | VTE | 19 | 6780 | 2.80 (1.79–4.39) | 1.71 (1.07–2.74) |
|  | No VTE | 1088 | 1,420,569 | 0.77 (0.72–0.81) |  |
| Liver, gallbladder, biliary tree | VTE | 6 | 6780 | 0.88 (0.40–1.97) | 3.33 (1.35–8.21) |
|  | No VTE | 155 | 1,420,569 | 0.11 (0.09–0.13) |  |
| Pancreas | VTE | 11 | 6780 | 1.62 (0.90–2.93) | 5.41 (2.84–10.34) |
|  | No VTE | 220 | 1,420,569 | 0.15 (0.14–0.18) |  |
| Lung | VTE | 11 | 6780 | 1.62 (0.90–2.93) | 2.71 (1.48–4.96) |
|  | No VTE | 460 | 1,420,569 | 0.32 (0.30–0.35) |  |
| Melanoma | VTE | 6 | 6780 | 0.88 (0.40–1.97) | 2.43 (1.08–5.48) |
|  | No VTE | 351 | 1,420,569 | 0.25 (0.22–0.27) |  |
| Breast^b^ | VTE | 10 | 2817 | 3.55 (1.91–6.60) | 1.16 (0.60–2.23) |
|  | No VTE | 1606 | 730,280 | 2.20 (2.09–2.31) |  |
| Uterus^b^ | VTE | 4 | 2817 | 1.42 (0.53–3.78) | 1.59 (0.59–4.28) |
|  | No VTE | 373 | 730,280 | 0.51 (0.46–0.57) |  |
| Ovarian^b^ | VTE | 6 | 2817 | 2.13 (0.96–4.74) | 7.64 (3.34–17.47) |
|  | No VTE | 182 | 730,280 | 0.25 (0.22–0.29) |  |
| Prostate^a^ | VTE | 33 | 3963 | 8.33 (5.92–11.71) | 1.01 (0.70–1.46) |
|  | No VTE | 2112 | 690,290 | 3.06 (2.93–3.19) |  |
| Kidney | VTE | 10 | 6780 | 1.47 (0.79–2.74) | 3.25 (1.52–6.97) |
|  | No VTE | 211 | 1,420,569 | 0.15 (0.13–0.17) |  |
| Bladder and urinary tract | VTE | 5 | 6780 | 0.74 (0.31–1.77) | 0.64 (0.21–2.01) |
|  | No VTE | 394 | 1,420,569 | 0.28 (0.25–0.31) |  |
| Brain | VTE | 4 | 6780 | 0.59 (0.22–1.57) | 1.96 (0.73–5.29) |
|  | No VTE | 337 | 1,420,569 | 0.24 (0.21–0.26) |  |
| Lymphoma | VTE | 4 | 6780 | 0.59 (0.22–1.57) | 0.97 (0.31–3.02) |
|  | No VTE | 361 | 1,420,569 | 0.25 (0.23–0.28) |  |
| Leukaemia | VTE | 7 | 6780 | 1.03 (0.49–2.17) | 3.06 (1.43–6.56) |
|  | No VTE | 233 | 1,420,569 | 0.16 (0.14–0.19) |  |
| Other haematological | VTE | 3 | 6780 | 0.44 (0.14–1.37) | 1.87 (0.59–5.88) |
|  | No VTE | 215 | 1,420,569 | 0.15 (0.13–0.17) |  |
| Unknown primary/metastatic | VTE | 12 | 6780 | 1.77 (1.01–3.12) | 7.64 (4.10–14.26) |
|  | No VTE | 183 | 1,420,569 | 0.13 (0.11–0.15) |  |
| Other cancers | VTE | 15 | 6780 | 2.21 (1.33–3.67) | 2.44 (1.40–4.25) |
|  | No VTE | 698 | 1,420,569 | 0.49 (0.46–0.53) |  |

* The associations are shown as hazard ratios with 95 % confidence intervals. A multivariable Cox proportional hazards regression model was used. In this analysis, all study participants (n=105,997) were included. Participants were followed for a total of 1,427,349 person years and 9822 participants were diagnosed with cancer during follow-up. Adjustment was made for age, sex, body mass index, smoking and education level. Venous thromboembolism was treated as a time-dependent covariate. Sex specific analyses were made for the associations between VTE and risk of sex-specific cancer types.

VTE, venous thromboembolism

^a^ In men only.

^b^ In women only.

**Appendix table 3** Baseline characteristics of participants with and without VTE during follow-up at first health examination (n=105,997)

|  | VTE during follow-up | No VTE during follow-up | P-value |
| --- | --- | --- | --- |
| Age, years | 51.8 (8.7) | 46.1 (9.2) | <0.001 |
| Female sex | 893 (45.1%) | 52,409 (50.4%) | <0.001 |
| Smoking |  |  |  |
| Never smoker | 837 (43.3%) | 50,339 (49.3%) | <0.001 |
| Ever smoker | 1095 (56.7%) | 51,733 (50.7%) |  |
| Education level |  |  |  |
| Secondary school or below | 1551 (80.7%) | 73,590 (72.0%) | <0.001 |
| Above secondary school | 372 (19.3%) | 28,618 (28.0%) |  |
| BMI, kg/m^2^ | 26.9 (4.2) | 25.8 (4.2) | <0.001 |

VTE, venous thromboembolism

Data are reported as n (%) or mean (standard deviation).

BMI, body mass index
